# Innovation intensity and perceived economic efficiency: the mediating role of workplace health support and the moderating role of age bias

**DOI:** 10.3389/fpsyg.2026.1815581

**Published:** 2026-07-20

**Authors:** Mengyu Lu, Bowen Lu, Zhi Zhan, Xinchen Leng

**Affiliations:** 1 School of Accounting and International Trade, Suzhou Institute of Trade & Commerce, Suzhou, China; 2 Faculty of Built Environment, University of Malaya, Kuala Lumpur, Malaysia; 3 School of Finance, Zhongnan University of Economics and Law, Wuhan, China; 4 Northeastern University, Seattle, WA, United States

**Keywords:** age bias, conservation of resources, innovation intensity, perceived economic efficiency, workplace health support

## Abstract

Rapid innovation and demographic change are reshaping how workplace efficiency is assessed. This study examines whether perceived innovation intensity is associated with perceived economic efficiency through workplace health support and whether age bias conditions this association. Drawing on conservation of resources theory and social categorization theory, we analyzed cross sectional survey data from 320 employed adults aged 45 to 55 years. The results showed that innovation intensity was positively associated with perceived economic efficiency (total association *B* = 0.635, *p* < 0.001; direct association *B* = 0.323, *p* < 0.001), and workplace health support accounted for part of this association. The indirect association was significant based on 5,000 bootstrap resamples [*B* = 0.312, 95% CI (0.248, 0.381)]. Age bias further moderated the association between innovation intensity and perceived economic efficiency, with the interaction term adding explanatory variance (Delta *R*-squared = 0.026, *p* < 0.01). Conditional indirect associations through workplace health support were significant at low, mean, and high levels of age bias, although the differences among these conditional effects were small. These findings suggest that perceived efficiency evaluations in innovation intensive workplaces are not shaped by innovation demands alone. Rather, they are linked to health supportive organizational resources and to the age related evaluation climate in which innovation related performance is interpreted.

## 1 Introduction

Innovation intensity has become a central feature of contemporary work organizations. Digital tools, process redesign, platform based coordination, and data driven performance systems have changed not only how employees work, but also how efficiency is evaluated. Efficiency is increasingly judged not only by output volume or short term cost reduction, but also by whether employees can adapt to new tools, maintain stable participation, absorb changing task requirements, and continue producing reliable outcomes under technological and procedural change. Innovation research shows that organizational performance depends on the adoption of new products, processes, and administrative changes within organizational routines (Damanpour, 1991; Damanpour and Evan, 1984). Innovation capability research further suggests that innovativeness, learning orientation, and organizational capability can improve responsiveness and business performance when new knowledge is translated into work practices (Calantone et al., 2002; Hult et al., 2004). Organizational innovativeness research also distinguishes innovation determinants, innovation types, and performance outcomes (Subramanian and Nilakanta, 1996; Wang and Ahmed, 2004). Human resource research similarly shows that performance outcomes depend on the conditions under which employees are asked to contribute, learn, and sustain effort (Delaney and Huselid, 1996; Jiang et al., 2012). These studies support the expectation that innovation intensity may be associated with higher efficiency, but they also imply that this relationship should not be treated as automatic. Innovation can improve coordination and resource use, but it can also increase uncertainty, workload, and demands on employees' physical, cognitive, and psychological resources.

This study focuses on workplace health support as a mechanism linking innovation intensity to perceived economic efficiency. Workplace health support refers to perceived organizational support for maintaining health, work capacity, and participation under changing work demands. It is distinct from individual health status because it captures the support environment surrounding employee health and work ability. The theoretical logic is grounded in conservation of resources theory, which argues that individuals seek to obtain, protect, and retain valued resources, and that stress occurs when resources are threatened, lost, or insufficient to meet demands (Hobfoll, 1989). Innovation intensity may provide better tools and clearer processes, but it may also require employees to invest time, attention, learning effort, and adaptive capacity. Workplace health support can help preserve employee resources, making innovation intensity more likely to correspond to sustained efficiency rather than fatigue, withdrawal, or reduced participation. This reasoning is consistent with perceived organizational support research (Eisenberger et al., 1986; Rhoades and Eisenberger, 2002), job demands and resources theory (Bakker and Demerouti, 2007; Schaufeli and Bakker, 2004), work design research (Hackman and Oldham, 1976; Parker et al., 2017), and studies linking workplace health practices or health shocks to performance outcomes (Lamm et al., 2007; Rettl et al., 2022).

The study also examines age bias as a boundary condition. Mid and late career employees may possess experience, task knowledge, and organizational memory, but they may also be subject to assumptions about adaptability, learning speed, technological responsiveness, or future contribution. Age related evaluation research shows that stereotypes about older workers often concern trainability, adaptability, productivity, and technological competence, although meta analytic evidence indicates that many negative assumptions about age and job performance are overstated or context dependent (Ng and Feldman, 2008, 2012; Posthuma and Campion, 2009; Truxillo et al., 2015). Research on age diversity climate also shows that organizational climates shape whether age differences become a source of inclusion or biased evaluation (Boehm et al., 2014; Kunze et al., 2011, 2013). Social categorization theory explains why age bias may become salient when performance standards are changing and information is incomplete (Tajfel and Turner, 1979). Age bias may therefore condition the association between innovation intensity and perceived efficiency. This does not mean that age bias is beneficial. Rather, biased evaluation climates may make innovation related performance more visible and more strongly connected to efficiency judgments. Using survey data from 320 employed adults aged 45 to 55 years, this study examines whether workplace health support explains the association between innovation intensity and perceived economic efficiency and whether age bias conditions this process.

The study makes three contributions. Theoretically, it extends conservation of resources theory to innovation-intensive aging workplaces by showing that workplace health support can operate as a resource pathway linking innovation intensity to perceived economic efficiency. Methodologically, it tests a moderated mediation model in which workplace health support explains part of the innovation-efficiency association and age bias conditions the strength of innovation-related efficiency judgments. Practically, it identifies a concrete organizational implication: innovation initiatives aimed at mid- and late-career employees should be paired with health-supportive work conditions and age-fair evaluation practices if efficiency gains are to be sustainable and equitable.

## 2 Literature review

### 2.1 Innovation intensity and perceived economic efficiency

Innovation intensity refers to the perceived extent to which a workplace emphasizes new tools, new procedures, continuous improvement, digital upgrading, and repeated adjustment of work routines. In many organizations, innovation is no longer an occasional strategic initiative, but a continuing condition of work. Employees are expected to learn new systems, modify routines, respond to changing performance indicators, and coordinate across redesigned processes. Under these conditions, innovation intensity can shape how employees understand both their own contribution and the organization's efficiency. Economic efficiency is also changing in meaning. Traditional indicators often emphasize output per unit of input, cost reduction, production volume, or resource utilization. These indicators remain important, but they do not fully capture efficiency in workplaces where systems and routines change frequently. In such contexts, efficiency also depends on whether employees can sustain performance while adapting to new tools and expectations. Thus, perceived economic efficiency in this study refers to employees' evaluation of whether resources, tasks, and work outcomes are managed effectively in their work setting. Innovation intensity may be positively associated with perceived economic efficiency because new tools and procedures can reduce duplication, accelerate communication, and make resource allocation more precise. Digital systems and redesigned workflows may also help employees identify problems earlier and respond more quickly. Repeated engagement with improvement processes may strengthen learning and adaptive capacity, allowing employees to solve problems and adjust to changing demands more effectively.

Prior research supports this expectation. Organizational innovation has been linked to performance because it allows firms to adapt structures, processes, and knowledge resources to changing environments (Damanpour and Evan, 1984; Hult et al., 2004). Reviews of organizational innovation also emphasize that innovation should be understood as a multidimensional organizational capability rather than a single technical output (Crossan and Apaydin, 2010). Research on innovation capability also shows that process and administrative innovation can improve responsiveness and resource use when employees understand and enact changes in daily work (Calantone et al., 2002; Jimenez-Jimenez and Sanz-Valle, 2011). At the same time, this relationship should not be treated as automatic. Innovation can create new demands, including unfamiliar systems, faster work cycles, and uncertain evaluation standards. From the perspective of conservation of resources theory, innovation intensity creates both resource opportunities and resource demands (Hobfoll, 1989). Employees' perceptions are therefore important because objective innovation indicators may not reveal how innovation is experienced in daily work. This perceptual approach is consistent with organizational climate and human resource research showing that experienced practices shape employee responses (Bowen and Ostroff, 2004; Nishii et al., 2008; Schneider et al., 2013). As a first step, innovation intensity should be positively associated with perceived economic efficiency when employees perceive innovation as improving work processes and performance outcomes.

Hypothesis 1 (H1). Innovation intensity is positively associated with perceived economic efficiency.

### 2.2 Workplace health support as a resource pathway

Workplace health support refers to perceived organizational support for maintaining health, work capacity, and participation under changing work demands. Systematic evidence suggests that workplace resources can support both employee wellbeing and performance outcomes (Nielsen et al., 2017). This construct does not refer to employees' individual health status. Rather, it captures whether employees perceive that the organization provides support, adjustment, responsiveness, and resources that help them continue working effectively. In innovation intensive workplaces, this distinction is important because the issue is not only whether employees are healthy, but whether the organization creates conditions that allow employees to sustain performance while adapting to change. Age-diverse workforce research also emphasizes the importance of work capacity and health-related support in later career stages (Harper, 2011). Conservation of resources theory provides the main theoretical basis for this mediating mechanism. According to this theory, people seek to protect valued resources and avoid resource loss, and work demands become problematic when they threaten or deplete resources faster than individuals can restore them (Hobfoll, 1989). Innovation intensity may generate resource pressure because employees must learn new procedures, update skills, and maintain output during transition. Workplace health support can reduce this pressure by helping employees preserve physical, psychological, and participation resources. When such support is available, employees may be better able to convert innovation demands into effective performance. Workplace health support can influence perceived economic efficiency by reducing disruption, improving adaptive participation, and preserving knowledge continuity. Employees who receive appropriate support are less likely to experience avoidable interruptions, withdrawal, or reduced capacity during organizational change. They may also be more willing to engage with new processes rather than resist them. This is particularly important for mid and late career workers, who may hold valuable organizational experience and task specific knowledge.

Prior research provides support for this argument. Perceived organizational support research shows that employees who believe their organization values their contributions and cares about their wellbeing are more likely to show commitment, effort, and reciprocal work behavior (Eisenberger et al., 1986; Rhoades and Eisenberger, 2002). Work ability and occupational health research likewise suggests that supportive work conditions help employees sustain participation when demands are high (Ilmarinen, 2001; Schaufeli and Bakker, 2004). Broader workplace health and performance research also indicates that health supportive practices can be linked to productivity and organizational performance, although the strength of evidence varies by context and measurement (Lamm et al., 2007). Research on employee health shocks further suggests that poorer employee health can reduce firm performance, supporting the view that health related capacity is not peripheral to organizational outcomes (Rettl et al., 2022). The argument is also compatible with occupational stress and work design models. Job demands and resources theory proposes that demands and resources jointly shape strain, motivation, and performance (Bakker and Demerouti, 2007), while work design research indicates that autonomy, feedback, and support influence how employees respond to complex or changing tasks (Hackman and Oldham, 1976; Parker et al., 2017). Thus, workplace health support is not merely an additional predictor of efficiency. It is a resource pathway through which innovation intensity may become associated with perceived economic efficiency. Because the data are perceptual, the mediation model should be interpreted as a relationship among employees' evaluations of their work environment. Employees who perceive higher innovation intensity may also perceive stronger organizational efforts to support work capacity, especially when innovation initiatives are accompanied by support programs, flexible adjustments, or supervisor responsiveness. These perceptions of workplace health support may then be associated with higher perceived economic efficiency.

Hypothesis 2 (H2). Workplace health support statistically mediates the association between innovation intensity and perceived economic efficiency.

### 2.3 Age bias in innovation-intensive evaluation

Age bias refers to perceived age related assumptions or differential evaluation in the workplace. It may include beliefs about adaptability, learning speed, technological ability, reliability, or future contribution that are tied to age rather than actual performance. Age bias can operate explicitly through discriminatory behavior, but it can also operate subtly through expectations and evaluation standards. In innovation intensive workplaces, age bias may become especially salient because innovation often emphasizes adaptability, speed, and technological responsiveness. The relevance of age bias does not depend on treating mid and late career employees as less adaptable. On the contrary, many workers in this age range possess extensive experience, problem solving ability, and organizational knowledge that can support innovation. The issue is that age related assumptions may influence how their performance is interpreted. For example, an employee's need for training may be viewed as normal adaptation for one group but as evidence of declining capability for another. Similarly, caution during implementation may be interpreted as careful risk management or as resistance to change depending on age related expectations. These interpretive differences can affect perceived efficiency. Age related evaluation research shows that stereotypes about older workers can concern adaptability, trainability, productivity, and technological competence (Posthuma and Campion, 2009; Ng and Feldman, 2012). At the same time, meta analytic evidence indicates that many negative assumptions about age and job performance are overstated or context dependent (Ng and Feldman, 2008; Truxillo et al., 2015). This makes age bias an evaluation climate issue rather than a simple reflection of ability.

Social categorization theory helps explain this moderating process. The theory suggests that people classify others into social categories and that these categories influence judgment, especially when information is incomplete or environments are uncertain (Tajfel and Turner, 1979). Innovation intensive settings often create uncertainty because tasks, tools, and performance criteria change. Under these conditions, evaluators may rely more heavily on simplified assumptions about who is likely to adapt quickly, learn new systems, or contribute to future performance. Age can become one such category. As a result, age bias may shape how innovation related behavior is evaluated and may moderate the relationship between innovation intensity and perceived economic efficiency. A negative moderation effect would suggest that age bias weakens the benefits of innovation by discouraging participation, undermining support, or devaluing contributions. A positive moderation effect, by contrast, would suggest that age bias makes innovation related performance cues more salient, strengthening the observed relationship between innovation intensity and efficiency evaluations. Importantly, a positive moderation effect would not mean that age bias is beneficial. It would mean that in biased evaluation climates, innovation intensity may become more closely tied to judgments of efficiency because employees and evaluators pay closer attention to adaptability and performance under change. This framing is consistent with research on age identity and subjective age, which suggests that age related self perceptions can influence work motivation, self efficacy, and performance related outcomes when age categories become salient in the work environment (Kooij et al., 2011; Rodriguez-Cifuentes et al., 2018). Research on age diversity climate further suggests that organizational climates can shape whether age differences become a resource or a source of bias (Boehm et al., 2014; Kunze et al., 2013). Therefore, the present study tests whether age bias conditions the association between innovation intensity and perceived economic efficiency.

Hypothesis 3 (H3). Age bias strengthens the positive association between innovation intensity and perceived economic efficiency, such that this association is more pronounced at higher levels of age bias. This hypothesis concerns evaluative salience and should not be read as implying that age bias is beneficial or desirable.

### 2.4 Conditional indirect effects through workplace health support

The preceding arguments suggest a moderated mediation model. Innovation intensity is expected to be associated with perceived economic efficiency partly through workplace health support, while age bias is expected to condition how innovation related performance is interpreted. These two processes may also be connected because age bias can shape both the perceived need for support and the efficiency value attached to that support. When age related assumptions are salient, innovation demands may make health support more visible or more necessary. Employees may become more attentive to whether the organization supports their capacity to adapt, particularly when adaptability, learning speed, and technological responsiveness are being evaluated through age related expectations. In this sense, age bias may affect the first stage of the mediation pathway by changing how innovation intensity is linked to perceived workplace health support.

Age bias may also shape the second stage of the mediation pathway. When age related assumptions are salient, workplace health support may become more strongly connected to efficiency evaluations because support helps employees demonstrate continued contribution under conditions where adaptability is being scrutinized. Alternatively, biased evaluation climates may weaken the value of support if employees' contributions are devalued regardless of available resources. Thus, the direction and size of the conditional indirect effect are empirical questions, but theory suggests that age bias should matter for the mediation pathway. Testing conditional indirect effects therefore moves beyond asking whether workplace health support mediates the innovation and efficiency relationship on average. It asks whether this resource pathway operates similarly across different age bias contexts. This logic is consistent with moderated mediation approaches in organizational research, which examine whether indirect effects depend on theoretically meaningful boundary conditions (Edwards and Lambert, 2007; Hayes, 2022). It is also aligned with calls to examine workplace aging as a contextual process shaped by organizational practices rather than only by chronological age (Bal et al., 2011). In the present study, age bias is therefore expected to condition the indirect association between innovation intensity and perceived economic efficiency through workplace health support.

Hypothesis 4 (H4). The indirect association of innovation intensity with perceived economic efficiency through workplace health support is conditional on age bias.

## 3 Materials and methods

### 3.1 Participants and procedure

The study examines how perceived innovation intensity is associated with perceived economic efficiency through workplace health support, and whether age bias conditions this relationship. A cross sectional survey design was used. The analytic sample consisted of 320 employed adults aged 45 to 55 years (*M* = 50.18, SD = 3.09). This age range was selected because mid and late career employees remain active participants in contemporary work organizations while age related assumptions about adaptability, learning speed, technological responsiveness, and continued work capacity may become salient. Eligible participants were required to be currently employed, to fall within the target age range, and to have sufficient workplace experience to evaluate innovation demands, health support resources, age related evaluation climates, and efficiency related outcomes. Participants came from multiple industry sectors, which allowed the analysis to include basic work context heterogeneity.

The available sample information indicates that respondents were drawn from multiple industry sectors and varied in age, gender, education, and tenure. Because recruitment was conducted through an anonymous online survey platform, detailed organization-level information such as firm size, ownership type, managerial level, and geographic distribution was not retained in a form that could be reported reliably. The findings should therefore be interpreted as applying most directly to mid- and late-career employees represented in this survey context rather than to a fully specified industry or regional population.

Data were collected through an online questionnaire, and participants were recruited through the professional survey platform Wenjuanxing. Before completing the questionnaire, all participants received an information sheet explaining the purpose of the study, the voluntary nature of participation, anonymity, confidentiality, and their right to withdraw before submission. Prior to the main survey, a pilot study involving 20 working adults was conducted to evaluate the clarity, comprehensibility, and contextual relevance of the survey instrument. Participants completed the preliminary questionnaire and were subsequently invited to provide feedback regarding item wording, interpretation, and overall readability. Based on this feedback, minor wording adjustments were made to improve readability and reduce potential ambiguity. The finalized questionnaire was subsequently used in the main survey. A total of 450 questionnaires were distributed to employed adults aged 45 to 55 years through online workplace networks and professional contacts. Of these, 368 questionnaires were returned, yielding an initial return rate of 81.8%. Data screening was then conducted before the final analysis. Responses were excluded if they met any of the following criteria: more than 10% missing responses on the focal variables, failure on the attention check item, identical responses across all scale items, patterned responses suggesting careless completion, duplicate submissions identified through repeated contact information or response records, or completion times that were unrealistically short relative to the questionnaire length. After applying these exclusion criteria, 48 responses were removed, and 320 valid responses were retained for analysis. The final effective response rate was 71.1% based on the total number of distributed questionnaires, and the valid response rate among returned questionnaires was 87.0%.

The study was approved by the School of Accounting and International Trade. All participants were informed of the purpose of the study before completing the questionnaire. Participation was voluntary and anonymous, and respondents were told that they could withdraw before submitting the questionnaire. No personally identifiable or sensitive information was collected. Electronic informed consent was obtained from all participants before data collection.

### 3.2 Measures

All focal variables were treated as perceptual survey constructs. This clarification is important because the study does not measure objective innovation investment, objective health expenditures, audited firm level efficiency, or observed discriminatory behavior. Instead, the questionnaire measured respondents' perceptions of innovation intensity, workplace health support, age bias, and perceived economic efficiency in their work settings. All items were scored on a seven point Likert type scale ranging from 1, strongly disagree, to 7, strongly agree. For each construct, item responses were averaged so that higher values reflected higher perceived innovation intensity, stronger perceived workplace health support, higher perceived economic efficiency, or stronger perceived age bias. No reverse coded items were used.

The item domains were adapted from established measurement traditions and revised for the present workplace context. Innovation intensity items were adapted from organizational innovation and innovation capability research, including work on organizational innovativeness, process innovation, innovation orientation, and innovation capability (Damanpour, 1991; Wang and Ahmed, 2004; Calantone et al., 2002), and were worded to capture employees' perceptions of new tools, new procedures, digital upgrading, continuous improvement, and repeated adjustment of work routines. Workplace health support items were adapted from perceived organizational support, work ability, and job demands and resources research (Eisenberger et al., 1986; Ilmarinen, 2001; Bakker and Demerouti, 2007), with wording focused on perceived organizational support for maintaining health, work capacity, and participation under changing work demands. Economic efficiency items were adapted from perceived organizational performance and efficiency research (Delaney and Huselid, 1996; Hult et al., 2004), and assessed respondents' evaluations of resource use, task completion, and work outcome effectiveness. Age bias items were adapted from workplace age stereotyping, age discrimination, and age diversity climate research (Finkelstein et al., 1995; Posthuma and Campion, 2009; Kunze et al., 2011), and captured perceived age related assumptions about adaptability, learning speed, technological responsiveness, and continued contribution. Industry turbulence was included as a perceptual work context control variable rather than a categorical industry label; its items captured perceived technological change, innovation pressure, competitive intensity, and efficiency related demands in the broader industry environment. All adapted items were reviewed for conceptual consistency before formal data collection rather than treated as verbatim replications of the original scales. The complete list of measurement items is provided in Appendix A.

The questionnaire was administered in Chinese. A translation and back translation procedure was used to ensure conceptual equivalence. The English item pool was first translated into Chinese by a bilingual researcher familiar with organizational and human resource management terminology. A second bilingual researcher independently translated the Chinese version back into English. Discrepancies between the original and back translated versions were discussed and resolved through reconciliation, with priority given to conceptual equivalence rather than literal wording.

### 3.3 Data analysis

The analysis proceeded in five steps. First, descriptive statistics and Pearson correlations were calculated for all core constructs. Second, because the study uses OLS regression rather than structural equation modeling, measurement quality was evaluated using indicators appropriate to this framework: Cronbach's alpha, mean inter-item correlations, corrected item-total correlations, and HTMT discriminant validity ratios. Composite reliability and average variance extracted are SEM-derived indices and were therefore not computed, as doing so without a confirmatory factor analysis would introduce a methodological inconsistency between the measurement evaluation framework and the analytic framework. Third, multiple regression models were used to test the direct association between innovation intensity and perceived economic efficiency. Fourth, mediation was tested using bootstrap confidence intervals for indirect effects. Fifth, moderation and conditional indirect effects were evaluated using a PROCESS Model 58 equivalent specification with 5,000 bootstrap resamples (Hayes, 2022; Preacher and Hayes, 2008).

For clarity, age bias was handled differently across models. In the bivariate and descriptive analyses, age bias was treated as a focal perceptual construct. In the direct-effect and mediation models, the main paths linked innovation intensity to workplace health support and perceived economic efficiency. In the moderation model, age bias was entered as a main predictor together with the interaction term between innovation intensity and age bias. In the robustness model, age bias was entered as an additional covariate to examine whether the associations involving innovation intensity and workplace health support remained stable after controls were included.

Age, gender, education, tenure, age bias, and industry sector were included in a robustness model to evaluate whether the focal associations changed after demographic and work context controls were added. Age and tenure were treated as continuous variables. Gender, education, and industry sector were entered using their coded categories. The main conclusions were evaluated by comparing the coefficients for innovation intensity and workplace health support before and after adding these controls. Because all focal variables were collected from the same respondents, procedural and statistical steps were used to reduce and assess common method bias. Procedurally, the survey was anonymous, participants were told that there were no right or wrong answers, and item order was arranged to reduce obvious hypothesis guessing. Statistically, Harman's single factor test was conducted. An unrotated exploratory factor analysis of the focal items showed that the first factor explained 29.9% of the total variance, below the commonly used 50% threshold. This result suggests that a single dominant method factor is unlikely to account for the observed relationships among the constructs. Nevertheless, because the study uses cross sectional self report data, common method bias cannot be fully ruled out. The findings are therefore interpreted as associational rather than causal.

## 4 Results

### 4.1 Dsiations

Table 1 reports the descriptive statistics for the key study variables. Innovation intensity and perceived economic efficiency were rated at moderately high levels, with mean scores of 4.519 and 4.536, respectively. Workplace health support averaged 4.060, indicating a moderate level of perceived organizational support for maintaining health, work capacity, and participation under changing work demands. Age bias also showed meaningful variation across respondents (*M* = 4.192, SD = 0.969), supporting its use as a moderator in subsequent analyses. The final analytic sample included 320 employed adults aged 45 to 55 years.

**Table 1:** Summary statistics for key study variables.

Variable	*N*	Mean	SD	Min	Max
Innovation intensity	320	4.519	0.885	2.50	7.00
Workplace health support	320	4.060	1.013	1.50	7.00
Perceived economic efficiency	320	4.536	0.898	2.00	7.00
Age bias	320	4.192	0.969	1.75	6.50
Industry turbulence	320	4.217	0.953	1.75	6.50

Table 2 reports the Pearson correlations among the core constructs. Innovation intensity was positively associated with perceived economic efficiency (*r* < 0.001) and workplace health support (*r* = 0.394, *p* < 0.001), indicating that respondents who perceived stronger innovation intensity also tended to report stronger workplace health support and higher perceived economic efficiency. Workplace health support showed the strongest bivariate association with perceived economic efficiency (r = 0.686, *p* < 0.001), providing preliminary support for its role as a potential mediating mechanism. Age bias was positively associated with innovation intensity (*r* = 0.326, *p* < 0.001), workplace health support (*r* = 0.319, *p* < 0.001), and perceived economic efficiency (*r* = 0.471, *p* < 0.001), supporting its inclusion as a theoretically relevant boundary condition. Industry context was also positively correlated with the focal constructs, but the correlations were modest to moderate, suggesting that industry related conditions should be controlled in subsequent robustness analyses rather than treated as a substitute for the focal explanatory variables.

**Table 2:** Pearson correlations among core constructs.

Variable	1	2	3	4	5
1. Innovation intensity	1.000				
2. Workplace health support	0.394^***^	1.000			
3. Perceived economic efficiency	0.609^***^	0.686^***^	1.000		
4. Age bias	0.326^***^	0.319^***^	0.471^***^	1.000	
5. Industry turbulence	0.199^***^	0.142^***^	0.320^***^	0.319^***^	1.000

^***^ *p* < 0.001.

### 4.2 Measurement quality and discriminant evidence

Table 3 reports scale reliability indicators and Cronbach's alpha values ranged from 0.792 to 0.836, mean inter-item correlations ranged from 0.487 to 0.560, and corrected item-total correlations were consistently acceptable. These results indicate satisfactory internal consistency across the short four-item scales.

**Table 3:** Scale reliability indicators.

Construct	Items	Cronbach's alpha	Mean inter-item r	Corrected item-total r
Innovation intensity	4	0.792	0.487	0.584–0.628
Workplace health support	4	0.830	0.550	0.616–0.703
Perceived economic efficiency	4	0.836	0.560	0.639–0.700
Age bias	4	0.830	0.549	0.617–0.681
Industry turbulence	4	0.812	0.519	0.615–0.643

Table 4 reports the HTMT ratios among the five constructs. All HTMT values were below the 0.90 threshold, with the largest value observed for workplace health support and perceived economic efficiency (HTMT = 0.824). These results support acceptable discriminant validity for the measurement structure while acknowledging that health support and efficiency remain theoretically related constructs.

**Table 4:** HTMT discriminant validity ratios.

Construct	1	2	3	4	5
1. Innovation intensity	1.000				
2. Workplace health support	0.485	1.000			
3. Perceived economic efficiency	0.748	0.824	1.000		
4. Age bias	0.402	0.384	0.566	1.000	
5. Industry turbulence	0.250	0.173	0.389	0.389	1.000

HTMT = heterotrait-monotrait ratio of correlations. Values below 0.90 indicate acceptable discriminant validity; values below 0.85 are preferable (Henseler et al., 2015).

### 4.3 Regression and mediation results

Table 5 reports the regression model predicting perceived economic efficiency. The model explained 60.7% of the variance in perceived economic efficiency (*R2* = 0.607, adjusted *R2* = 0.604). Innovation intensity was positively associated with perceived economic efficiency (*B* = 0.407, SE = 0.039, *t* = 10.459, *p* < 0.001), supporting H1. Workplace health support also showed a positive association with perceived economic efficiency (*B* = 0.469, SE = 0.034, *t* = 13.800, *p* < 0.001). These results indicate that innovation intensity remained significantly associated with perceived efficiency after workplace health support was included in the model.

**Table 5:** Multiple regression model predicting perceived economic efficiency.

Variable	*B*	SE	*T*	Sig.
Constant	0.797	0.178	4.468	^***^
Innovation Intensity	0.407	0.039	10.459	^***^
Workplace Health Support	0.469	0.034	13.800	^***^

Model R2 = 0.607; adjusted R2 = 0.604. Cohen's f2 effect sizes were 0.345 for Innovation Intensity and 0.601 for workplace health support (Cohen, 1988). ^***^ *p* < 0.001.

Table 6 reports the mediation analysis linking innovation intensity to perceived economic efficiency through workplace health support. Conceptually, the a path represents the association between innovation intensity and workplace health support, the b path represents the association between workplace health support and perceived economic efficiency after accounting for innovation intensity, the c path represents the total association between innovation intensity and perceived economic efficiency, and the c' path represents the direct association after workplace health support is included. The total association of innovation intensity with perceived economic efficiency was positive and significant [*B* = 0.635, 95% CI (0.555, 0.713)]. After workplace health support was included, the direct association remained significant [*B* = 0.323, 95% CI (0.237, 0.407)]. The indirect association through workplace health support was also significant [*B* = 0.312, 95% CI (0.248, 0.381)], indicating partial statistical mediation and supporting H2. These results suggest that innovation intensity is related to perceived economic efficiency both directly and indirectly through workplace health support. Because the data are cross sectional and perceptual, the mediation should be interpreted as a theoretically consistent pattern of associations rather than as evidence that innovation intensity causally determines workplace health support or perceived economic efficiency.

**Table 6:** Bootstrap decomposition of total, direct, and indirect effects.

Path	Effect	Boot SE	95% CI	Sig.
Total effect (c)	0.635	0.041	[0.555, 0.713]	^***^
Direct effect (c')	0.323	0.043	[0.237, 0.407]	^***^
Indirect effect (a^*^b)	0.312	0.034	[0.248, 0.381]	^***^

Bootstrap confidence intervals were estimated with 5,000 resamples. ^***^ *p* < 0.001.

### 4.4 Conditional effects under age bias

Table 7 reports the moderation model predicting perceived economic efficiency. Innovation intensity remained positively associated with perceived economic efficiency (*B* = 0.503, SE = 0.043, *p* < 0.001). Age bias also showed a positive association with perceived economic efficiency (*B* = 0.258, SE = 0.041, *p* < 0.001). The interaction between innovation intensity and age bias was positive and significant (*B* = 0.152, SE = 0.045, *p* < 0.01), supporting H3.

**Table 7:** Interaction model with age bias as moderator.

Variable	*B*	SE	*T*	Sig.
Constant	3.950	0.174	22.674	^***^
Innovation intensity	0.503	0.043	11.562	^***^
Age bias	0.258	0.041	6.254	^***^
Industry turbulence	0.129	0.040	3.199	^**^
Innovation intensity x age bias	0.152	0.045	3.395	^**^

Predictors were mean centered before the interaction term was created. Model *R2* = 0.489; adjusted *R2* = 0.483. The interaction term (Innovation Intensity × Age Bias) added Δ*R*^2^ = 0.026 (*p* < 0.01) over the model without the interaction. ^**^ *p* < 0.01. ^***^ *p* < 0.001.

### 4.5 Conditional indirect effects under age bias

Table 8 reports the conditional indirect effects of innovation intensity on perceived economic efficiency through workplace health support at low, mean, and high levels of age bias, controlling for age and gender. The indirect effect was significant at all three levels: low age bias [*B* = 0.107, 95% CI (0.053, 0.169)], mean age bias [B = 0.158, 95% CI (0.105, 0.216)], and high age bias [*B* = 0.218, 95% CI (0.123, 0.323)]. The conditional indirect effect increased consistently across age bias levels, and the contrast between mean and low age bias was significant [*B* = 0.051, 95% CI (0.001, 0.100)]. However, the contrasts between high and mean and between high and low age bias included zero, and the linear component of the moderated mediation index, while positive, also narrowly included zero [*B* = 0.057, 95% CI (−0.003, 0.120)]. Taken together, these results indicate that workplace health support remained a significant mediating mechanism across all age bias contexts and that the indirect effect tended to strengthen as age bias increased, but the moderated mediation should be interpreted as modest and cautiously supported rather than as robust evidence of a clear boundary condition. H4 is therefore interpreted as receiving trend-level support.

**Table 8:** Conditional indirect effects and moderated mediation tests.

Panel/effect	Estimate	Boot SE	95% CI lower	95% CI upper
Panel A. conditional indirect effects				
Low age bias (-1 SD)	0.107	0.030	0.053	0.169
Mean age bias	0.158	0.028	0.105	0.216
High age bias (+1 SD)	0.218	0.051	0.123	0.323
Panel B. bootstrap contrasts				
Mean—low	0.051	0.025	0.001	0.100
High—mean	0.060	0.035	−0.008	0.133
High—low	0.111	0.060	−0.007	0.233
Panel C. moderated mediation index components				
Linear component	0.057	0.031	−0.003	0.120
Quadratic component	0.005	0.006	−0.006	0.018

Bootstrap confidence intervals were estimated with 5,000 resamples. Age and gender were included as control variables. The conditional indirect effect refers to the association between innovation intensity and perceived economic efficiency through workplace health support at different levels of age bias. Because the Model 58 specification allows moderation of both the first-stage and second-stage paths, the moderated mediation function is nonlinear; therefore, both linear and quadratic index components are reported.

### 4.6 Collinearity and robustness checks

Table 9 reports the multicollinearity diagnostics for the predictors included in the regression models. All VIF values were below 2.0, ranging from 1.182 to 1.650, and all tolerance values exceeded 0.60. These results indicate that multicollinearity was not a serious concern in the main regression model. In the interaction specification, VIF values also remained acceptable after the component variables were mean centered, ranging from 1.138 to 1.645. Overall, the diagnostics suggest that the estimated regression coefficients were not substantially affected by collinearity among the predictors.

**Table 9:** Multicollinearity diagnostics for predictors.

Predictor	VIF	Tolerance	Assessment
Innovation intensity	1.557	0.642	Acceptable
Workplace health support	1.650	0.606	Acceptable
Age bias	1.216	0.823	Acceptable
Industry turbulence	1.182	0.846	Acceptable

VIF = variance inflation factor. Predictors were assessed in the regression models only. VIF values below 5 and tolerance values above 0.20 are generally considered acceptable.

Table 10 reports the regression model predicting perceived economic efficiency after adding demographic and work context controls. Innovation intensity remained positively associated with perceived economic efficiency (*B* = 0.318, SE = 0.042, *t* = 7.524, *p* < 0.001), and workplace health support also remained positively associated with perceived economic efficiency (*B* = 0.449, SE = 0.040, *t* = 11.351, *p* < 0.001). These results indicate that the main associations were robust after controlling for age, gender, education, tenure, age bias, and industry turbulence. Among the control variables, industry turbulence showed a small but significant positive association with perceived economic efficiency (*B* = 0.082, SE = 0.035, *t* = 2.317, *p* < 0.05), suggesting that broader industry conditions may also be relevant to perceived efficiency evaluations. Age, gender, education, tenure, and age bias were not significant predictors in this controlled model. Overall, the findings support the robustness of the central relationship among innovation intensity, workplace health support, and perceived economic efficiency.

**Table 10:** Regression model predicting perceived economic efficiency with controls.

Variable	B	SE	*T*	**Sig**.
Constant	0.201	0.671	0.300	
Innovation intensity	0.318	0.042	7.524	^***^
Workplace health support	0.449	0.040	11.351	^***^
Age bias	0.043	0.036	1.209	
Industry turbulence	0.082	0.035	2.317	^*^
Age	0.014	0.012	1.159	
Gender	0.032	0.075	0.430	
Education	−0.060	0.040	−1.513	
Tenure	−0.003	0.006	−0.454	

^*^ *p* < 0.05. ^***^ *p* < 0.001.

## 5 Discussion

### 5.1 Main findings and theoretical implications

The present study examined whether innovation intensity is associated with perceived economic efficiency, whether workplace health support accounts for part of this association, and whether age bias changes the strength of this process. The findings generally support the proposed model. H1 was supported, as innovation intensity was positively associated with perceived economic efficiency. H2 was also supported, as workplace health support statistically mediated this association. H3 was supported by the significant interaction between innovation intensity and age bias. H4 received cautious support because the conditional indirect associations through workplace health support were significant at low, mean, and high levels of age bias, although the observed differences among these conditional effects were small. Together, these findings suggest that perceived efficiency evaluations in innovation intensive workplaces are not shaped by innovation demands alone. They are also linked to the organizational resources that help employees maintain work capacity and to the age related evaluation climate in which innovation related performance is interpreted. The observed effect sizes were meaningful within this perceptual survey context, but they should not be interpreted as changes in audited productivity, financial returns, or objective organizational performance. Rather, they indicate strong alignment among respondents' perceptions of innovation demands, health support resources, and perceived economic efficiency.

Theoretically, these findings extend conservation of resources theory by showing that workplace health support can function as a resource pathway associated with perceived economic efficiency in innovation intensive work. Innovation intensive work may improve perceived work processes, but it can also create learning demands, adjustment pressure, and uncertainty in performance expectations. Health supportive organizational resources may help employees preserve the physical, psychological, and participation resources needed to adapt, remain involved, and sustain performance under changing work demands. The findings also clarify the role of age bias in innovation intensive workplaces. The positive interaction between innovation intensity and age bias should not be interpreted as evidence that age bias is useful, fair, or desirable. Rather, it suggests that age bias increases the evaluative salience of innovation related performance. When age related assumptions are more salient, innovation intensity may become more closely tied to judgments about adaptability, technological responsiveness, learning speed, and continued contribution. This is an evaluative-salience interpretation, not a justification for age discrimination. Age bias can damage fairness, motivation, engagement, psychological safety, and access to development opportunities, even when it makes innovation related behavior more visible in performance evaluations. Overall, the study contributes to innovation management, workplace health, and age at work research by showing that perceived economic efficiency is connected not only to innovation intensity itself, but also to the support resources and social evaluation processes surrounding innovation.

### 5.2 Practical implications

The findings offer practical implications for organizations pursuing innovation while managing employee sustainability and workforce aging. First, innovation initiatives should not be implemented as purely technical or procedural changes. New tools, digital systems, redesigned workflows, and continuous improvement programs may be associated with higher perceived efficiency, but their value depends partly on whether employees have the resources needed to adapt and remain effective. Organizations should therefore pair innovation programs with workplace health support, including reasonable workload planning, accessible training, flexible task adjustment, recovery opportunities, supervisor responsiveness, and health related accommodations. Workplace health support should be treated as part of the innovation infrastructure rather than as a separate welfare activity.

Second, organizations should monitor how age related assumptions enter performance evaluation during innovation change. The positive moderating role of age bias does not mean that age bias improves efficiency. Rather, it indicates that when age related evaluation cues are salient, innovation intensity becomes more closely tied to judgments about adaptability, technological responsiveness, learning speed, and continued contribution. This creates a practical risk for mid and late career employees, whose performance may be interpreted through assumptions about their age group rather than their actual contribution. Managers should therefore use transparent evaluation standards, provide equal access to reskilling opportunities, and avoid interpreting training needs, cautious decision making, or slower adjustment to unfamiliar systems as evidence of declining ability. Sustainable innovation requires not only stronger innovation intensity, but also healthier support systems and fairer evaluation climates.

### 5.3 Limitations and future research

Several limitations should be acknowledged. First, the study used a cross sectional survey design, so the findings should be interpreted as associations rather than causal effects. Although the proposed model is theoretically grounded, the data cannot establish whether innovation intensity leads to later changes in workplace health support or perceived economic efficiency. Future research should use longitudinal, multi wave, or quasi experimental designs to examine whether changes in innovation intensity are followed by changes in support resources and efficiency outcomes over time. Second, all focal variables were measured through self report perceptions from the same respondents at the same time. This approach is appropriate because the study focuses on employees' evaluations of innovation intensity, workplace health support, age bias, and perceived economic efficiency, but it creates common method bias concerns. Procedural safeguards and Harman's single factor test provide only preliminary reassurance and cannot fully rule out inflated associations due to shared source, response style, social desirability, or transient mood. Future studies should combine employee surveys with supervisor ratings, administrative records, objective productivity indicators, absenteeism data, documented health support practices, or time separated measurements.

Third, the dependent variable captures perceived economic efficiency rather than audited financial performance or objective operational output. The results therefore speak to how employees evaluate efficiency in their work setting, not to verified firm level economic performance. This distinction is especially important because workplace health support and perceived economic efficiency were strongly associated. Conceptually, workplace health support refers to perceived organizational resources that help employees maintain health, work capacity, and participation, whereas perceived economic efficiency refers to respondents' judgments about resource use, process effectiveness, and productive use of organizational inputs. The constructs are related but not interchangeable. Future research could test whether the same model predicts objective outcomes such as unit productivity, cost reduction, error rates, project completion efficiency, or financial indicators. Fourth, although industry turbulence was included as a control variable, future research should examine richer organizational conditions, including firm size, job role, managerial status, technology intensity, organization type, formal innovation investment, and geographic distribution. Finally, it should be noted that innovation intensity produced the lowest internal consistency among the five constructs (Cronbach's alpha = 0.792), which, while above the conventional 0.70 threshold, was the weakest of the five scales. Future research should invest in refining and extending the innovation intensity items to improve measurement precision, particularly if the construct is deployed in settings with greater heterogeneity in innovation practices.

## 6 Conclusion

This study examined how innovation intensity, workplace health support, and age bias are associated with perceived economic efficiency among employed adults aged 45 to 55 years. The findings show that innovation intensity is positively associated with perceived economic efficiency and that workplace health support partially mediates this relationship. These results suggest that innovation may be linked to efficiency not only through direct improvements in work processes and coordination, but also through health supportive organizational resources that help employees sustain participation, preserve work capacity, and adapt to changing demands. The findings also show that age bias conditions the relationship between innovation intensity and perceived economic efficiency. The positive interaction should not be interpreted as evidence that age bias is beneficial. Rather, it suggests that when age related evaluation cues are more salient, innovation intensity becomes more strongly connected to efficiency judgments. This pattern highlights the importance of fair performance standards, transparent evaluation criteria, and health supportive management practices in innovation intensive workplaces. Overall, the study contributes to innovation management, workplace health, and age at work research by showing that perceived efficiency outcomes are associated with both resource support mechanisms and age related evaluation contexts.

## Data Availability

The raw data supporting the conclusions of this article will be made available by the authors, without undue reservation.

## Appendix A. measurement items

All items were measured on a seven-point Likert scale ranging from 1 (strongly disagree) to 7 (strongly agree).

Innovation Intensity (II)

II01. My organization regularly introduces new tools and processes that I am expected to learn and adopt.
II02. I am expected to continuously seek improvements in how I carry out my work.
II03. Innovation is an important part of my organization's daily operations.
II04. My organization frequently introduces new methods or practices to enhance performance.

Workplace Health Support (HS)

HS01. My organization provides adequate support for my health and wellbeing at work.
HS02. Management takes employee health concerns seriously.
HS03. I have access to resources that support my physical and psychological wellbeing at work.
HS04. My organization actively promotes a healthy work environment.

Perceived Economic Efficiency (PEE)

PEE01. I feel that resources in my workplace are used efficiently.
PEE02. I perceive that work processes in my organization are managed in a cost-effective manner.
PEE03. In my view, my organization achieves a high level of operational efficiency.
PEE04. I feel that available resources are utilized productively to achieve organizational goals.

Age Bias (AB)

AB01. In my workplace, older employees are sometimes viewed as less adaptable to organizational change.
AB02. In my workplace, employee age influences how work performance is evaluated.
AB03. In my workplace, older workers may face disadvantages in performance-related judgments.
AB04. In my workplace, age-related stereotypes can affect workplace decisions.

Industry Turbulence (IT)

IT01. Changes in my industry occur rapidly.
IT02. Customer demands and market conditions in my industry frequently change.
IT03. Competitive conditions in my industry are highly dynamic.
IT04. Organizations in my industry must adapt continuously to external changes.

## References

[B1] Bakker A. B. Demerouti E. (2007). The job demands-resources model: state of the art. J. Manag. Psychol. 22, 309–328. doi: 10.1108/02683940710733115

[B2] Bal A. C. Reiss A. E. B. Rudolph C. W. Baltes B. B. (2011). Examining positive and negative perceptions of older workers: a meta-analysis. J. Gerontol. B Psychol. Sci. Soc. Sci. 66B, 687–698. doi: 10.1093/geronb/gbr056 21719634

[B3] Boehm S. A. Kunze F. Bruch H. (2014). Spotlight on age-diversity climate: the impact of age-inclusive HR practices on firm-level outcomes. Pers. Psychol. 67, 667–704. doi: 10.1111/peps.12047

[B4] Bowen D. E. Ostroff C. (2004). Understanding HRM-firm performance linkages: the role of the strength of the HRM system. Acad. Manag. Rev. 29, 203–221. doi: 10.5465/amr.2004.12736076

[B5] Calantone R. J. Cavusgil S. T. Zhao Y. (2002). Learning orientation, firm innovation capability, and firm performance. Ind. Mark. Manag. 31, 515–524. doi: 10.1016/S0019-8501(01)00203-6

[B6] Cohen J. (1988). Statistical Power Analysis for the Behavioral Sciences 2nd Edn. Hillsdale, NJ: Lawrence Erlbaum Associates.

[B7] Crossan M. M. Apaydin M. (2010). A multi-dimensional framework of organizational innovation: a systematic review of the literature. J. Manag. Stud. 47, 1154–1191. doi: 10.1111/j.1467-6486.2009.00880.x

[B8] Damanpour F. (1991). Organizational innovation: a meta-analysis of effects of determinants and moderators. Acad. Manag. J. 34, 555–590. doi: 10.2307/256406

[B9] Damanpour F. Evan W. M. (1984). Organizational innovation and performance: the problem of organizational lag. Adm. Sci. Q. 29, 392–409. doi: 10.2307/2393031

[B10] Delaney J. T. Huselid M. A. (1996). The impact of human resource management practices on perceptions of organizational performance. Acad. Manag. J. 39, 949–969. doi: 10.2307/256718

[B11] Edwards J. R. Lambert L. S. (2007). Methods for integrating moderation and mediation: a general analytical framework using moderated path analysis. Psychol. Methods 12, 1–22. doi: 10.1037/1082-989X.12.1.1 17402809

[B12] Eisenberger R. Huntington R. Hutchison S. Sowa D. (1986). Perceived organizational support. J. Appl. Psychol. 71, 500–507. doi: 10.1037/0021-9010.71.3.500

[B13] Finkelstein L. M. Burke M. J. Raju N. S. (1995). Age discrimination in simulated employment contexts: an integrative analysis. J. Appl. Psychol. 80, 652–663. doi: 10.1037/0021-9010.80.6.652

[B14] Hackman J. R. Oldham G. R. (1976). Motivation through the design of work: test of a theory. Organ. Behav. Hum. Perform. 16, 250–279. doi: 10.1016/0030-5073(76)90016-7

[B15] Harper S. (2011). “Health and wellbeing in older workers: Capacity change with age,” in Managing an age-diverse workforce, eds. E. Parry & S. Tyson (Basingstoke: Palgrave Macmillan), 206–220 doi: 10.1057/9780230299115_13

[B16] Hayes A. F. (2022). Introduction to mediation, Moderation, and Conditional Process Analysis: A Regression-Based Approach 3rd Edn. New York, NY: Guilford Press.

[B17] Henseler J. Ringle C. M. Sarstedt M. (2015). A new criterion for assessing discriminant validity in variance-based structural equation modeling. J. Acad. Mark. Sci. 43, 115–135. doi: 10.1007/s11747-014-0403-8

[B18] Hobfoll S. E. (1989). Conservation of resources: a new attempt at conceptualizing stress. Am. Psychol. 44, 513–524. doi: 10.1037/0003-066X.44.3.513 2648906

[B19] Hult G. T. M. Hurley R. F. Knight G. A. (2004). Innovativeness: Its antecedents and impact on business performance. Ind. Mark. Manag. 33, 429–438. doi: 10.1016/j.indmarman.2003.08.015

[B20] Ilmarinen J. (2001). Aging workers. Occup. Environ. Med. 58, 546–552. doi: 10.1136/oem.58.8.546 11452053 PMC1740170

[B21] Jiang K. Lepak D. P. Hu J. Baer J. C. (2012). How does human resource management influence organizational outcomes? A meta-analytic investigation of mediating mechanisms. Acad. Manag. J. 55, 1264–1294. doi: 10.5465/amj.2011.0088

[B22] Jimenez-Jimenez D. Sanz-Valle R. (2011). Innovation, organizational learning, and performance. J. Bus. Res. 64, 408–417. doi: 10.1016/j.jbusres.2010.09.010

[B23] Kooij D. T. A. M. de Lange A. H. Jansen P. G. W. Kanfer R. Dikkers J. S. E. (2011). Age and work-related motives: results of a meta-analysis. J. Organ. Behav. 32, 197–225. doi: 10.1002/job.665

[B24] Kunze F. Boehm S. A. Bruch H. (2011). Age diversity, age discrimination climate, and performance consequences. J. Appl. Psychol. 96, 264–290. doi: 10.1002/job.698

[B25] Kunze F. Boehm S. A. Bruch H. (2013). Organizational performance consequences of age diversity: inspecting the role of diversity-friendly HR policies and top managers' negative age stereotypes. J. Manag. Stud. 50, 413–442. doi: 10.1111/joms.12016

[B26] Lamm F. Massey C. Perry M. (2007). Is there a link between workplace health and safety and firm performance and productivity? N. Z. J. Employ. Relat. 32, 72–86.

[B27] Ng T. W. H. Feldman D. C. (2008). The relationship of age to ten dimensions of job performance. J. Appl. Psychol. 93, 392–423. doi: 10.1037/0021-9010.93.2.392 18361640

[B28] Ng T. W. H. Feldman D. C. (2012). Evaluating six common stereotypes about older workers with meta-analytical data. Pers. Psychol. 65, 821–858. doi: 10.1111/peps.12003

[B29] Nielsen K. Nielsen M. B. Ogbonnaya C. Kansala M. Saari E. Isaksson K. (2017). Workplace resources to improve both employee wellbeing and performance: a systematic review and meta-analysis. Work Stress 31, 101–120. doi: 10.1080/02678373.2017.1304463

[B30] Nishii L. H. Lepak D. P. Schneider B. (2008). Employee attributions of the why of HR practices: their effects on employee attitudes and behaviors, and customer satisfaction. Pers. Psychol. 61, 503–545. doi: 10.1111/j.1744-6570.2008.00121.x

[B31] Parker S. K. Morgeson F. P. Johns G. (2017). 100 years of work design research: looking back and looking forward. J. Appl. Psychol. 102, 403–420. doi: 10.1037/apl0000106 28182465

[B32] Posthuma R. A. Campion M. A. (2009). Age stereotypes in the workplace: common stereotypes, moderators, and future research directions. J. Manage. 35, 158–188. doi: 10.1177/0149206308318617

[B33] Preacher K. J. Hayes A. F. (2008). Asymptotic and resampling strategies for assessing and comparing indirect effects in multiple mediator models. Behav. Res. Methods 40, 879–891. doi: 10.3758/BRM.40.3.879 18697684

[B34] Rettl D. A. Schandlbauer A. Trandafir M. (2022). Employee health and firm performance (IZA Discussion Paper No. 15147). Bonn: IZA Institute of Labor Economics. doi: 10.2139/ssrn.4022672

[B35] Rhoades L. Eisenberger R. (2002). Perceived organizational support: a review of the literature. J. Appl. Psychol. 87, 698–714. doi: 10.1037/0021-9010.87.4.698 12184574

[B36] Rodriguez-Cifuentes F. Farfan J. Topa G. (2018). Older worker identity and job performance: the moderator role of subjective age and self-efficacy. Int. J. Environ. Res. Public Health 15:2731. doi: 10.3390/ijerph15122731 30514003 PMC6313778

[B37] Schaufeli W. B. Bakker A. B. (2004). Job demands, job resources, and their relationship with burnout and engagement. J. Organ. Behav. 25, 293–315. doi: 10.1002/job.248

[B38] Schneider B. Ehrhart M. G. Macey W. H. (2013). Organizational climate and culture. Annu. Rev. Psychol. 64, 361–388. doi: 10.1146/annurev-psych-113011-143809 22856467

[B39] Subramanian A. Nilakanta S. (1996). Organizational innovativeness: exploring the relationship between organizational determinants of innovation, types of innovations, and measures of organizational performance. Omega . 24, 631–647. doi: 10.1016/S0305-0483(96)00031-X

[B40] Tajfel H. Turner J. C. (1979). “An integrative theory of intergroup conflict,” in The Social Psychology of Intergroup Relations, eds., W. G. Austin & S. Worchel (Monterey, CA: Brooks/Cole), 33–47.

[B41] Truxillo D. M. Cadiz D. M. Hammer L. B. (2015). Supporting the aging workforce: a review and recommendations for workplace intervention research. Annu. Rev. Organ. Psychol. Organ. Behav. 2, 351–381. doi: 10.1146/annurev-orgpsych-032414-111435

[B42] Wang C. L. Ahmed P. K. (2004). The development and validation of the organizational innovativeness construct using confirmatory factor analysis. Eur. J. Innov. Manag. 7, 303–313. doi: 10.1108/14601060410565056

